# A network analysis of facial and vocal emotion recognition deficits in schizophrenia

**DOI:** 10.3389/fpsyt.2025.1598026

**Published:** 2025-05-23

**Authors:** Wenxuan Zhao, Qi Zhang, Long Gao, Ning Fan, Yajun Yun, Jiaqi Song, Yunhe Ji, Yongqian Wang, Meng Zhang, Fude Yang, Shuping Tan

**Affiliations:** ^1^ Peking University Huilonguan Clinical Medical School, Beijing Huilongguan Hospital, Beijing, China; ^2^ Wuxi Mental Health Center, Wuxi, China; ^3^ Changping Labroray, Beijing, China; ^4^ Yantai Psychological Rehabilitation Hospital, Yantai, China

**Keywords:** schizophrenia, emotion recognition, neuropsychological tests, principal component analysis, network analysis

## Abstract

**Introduction:**

Facial and vocal emotion recognition deficits are common in individuals with schizophrenia.

**Methods:**

In this observational, single-center study, 106 patients with schizophrenia (SCZ) and 118 age- and sex-matched healthy controls underwent cognitive and emotional function assessments. The Temporal Experience of Pleasure Scale (TEPS), Personal and Social Performance Scale, Positive and Negative Symptom Scale, and Brief Negative Symptom Scale were used to evaluate psychotic symptoms in the SCZ group. Participants were assessed using the MATRICS Consensus Cognitive Battery (MCCB), the Positive and Negative Syndrome Scale, and emotion recognition tests involving 42 facial and 42 vocal emotional tasks.

**Results:**

The SCZ group had significant impairments in facial and vocal emotion recognition, with lower accuracy across all emotional categories. Mean scores in the SCZ group were significantly lower than those in the control group (facial, 23.55 ± 7.10 vs. 31.86 ± 5.16; vocal, 18.64 ± 9.48 vs. 29.42 ± 5.01, respectively; p<0.001). Emotion recognition deficits and demographic or clinical characteristics were not significantly correlated. Network analysis revealed strong intercorrelations among different cognitive domains, linking MCCB performance to emotion recognition abilities (r>0.9; p<0.001). Integration of tests of cognitive function (MCCB, area under the curve [AUC]=91.90%, p<0.01), emotion recognition abilities (facial, AUC=82.56%; vocal, AUC=82.82%; p<0.01), and TEPS (AUC=91.13%, p<0.01) proved useful for distinguishing patients with schizophrenia from healthy individuals.

**Discussion:**

These findings underscore the importance of emotion recognition impairments in schizophrenia and their strong association with cognitive deficits. Future interventions should focus on targeted cognitive and affective training strategies. Incorporating multimodal assessments into clinical evaluations may enhance diagnostic accuracy.

## 1 Introduction

Schizophrenia is a severe mental disorder characterized by significant impairments in emotion recognition, particularly in accurately identifying others’ emotions (1). Studies consistently demonstrate that individuals with schizophrenia exhibit lower accuracy in emotion recognition compared to healthy individuals, which may contribute to difficulties in interpersonal interactions, ultimately affecting social functioning and quality of life (2, 3). Therefore, investigating the emotion recognition abilities of individuals with schizophrenia holds substantial theoretical and clinical significance as it aids in elucidating the nature of their social cognitive dysfunction and informing the development of effective treatment strategies.

Individuals with schizophrenia experience pronounced difficulties in identifying, naming, and distinguishing emotions in facial expressions, particularly negative emotions such as sadness, fear, and anger (4). Research examining the relationship between clinical symptoms and facial emotion recognition suggests associations with both positive and negative symptomatology (5). Moreover, longitudinal studies indicate that these deficits are present in patients with first-episode psychosis and individuals with chronic illness (6). Notably, patients with schizophrenia in remission exhibit persistent deficits in emotion labeling and emotional intensity prediction tasks, even when clinical symptoms are stabilized (7). In addition to facial emotion recognition, deficits in voice emotion recognition represent another critical area of impairment in patients with schizophrenia (8). Studies indicate that individuals with schizophrenia also demonstrate significant impairments in recognizing vocal emotions, likely attributable to abnormalities in the neural networks responsible for processing emotional information (9). These deficits not only hinder patients’ comprehension of others’ emotions but may also exacerbate social interaction difficulties.

Cognitive impairments in schizophrenia extend across multiple domains, including difficulties in emotion recognition (10). Research utilizing the MATRICS Consensus Cognitive Battery (MCCB) has consistently shown that individuals with schizophrenia underperform in various cognitive domains, including attention, working memory, verbal learning and memory, visual learning and memory, reasoning and problem-solving, and social cognition. These cognitive deficits are closely linked to social dysfunction, further impairing overall functioning and quality of life (11). Furthermore, a significant association has been identified between these cognitive impairments and deficits in emotion recognition (12), suggesting an intricate interplay between cognitive dysfunction and social cognitive deficits in patients with schizophrenia.

However, some studies have failed to demonstrate significant deterioration in emotion recognition, likely due to methodological heterogeneity. Differences in study design, including variations in patient selection criteria, assessment tools for facial recognition, and coexisting cognitive impairments (eg, memory deficits), may contribute to these discrepancies (13). Additionally, cultural differences in facial recognition and visual perception necessitate cross-cultural investigations to determine the universality of these findings and their relationship with psychopathology in diverse populations (3).

Given these complexities, a comprehensive approach that simultaneously examines facial emotion recognition, voice emotion recognition, and cognitive functioning (eg, MCCB assessments) is essential. A joint investigation of these domains can enable a more thorough evaluation of patients’ social cognitive abilities, elucidate the interplay between cognitive domains, and enhance the understanding of the relationship between emotion recognition deficits and broader cognitive impairments. By integrating cognitive function assessments, such as the MCCB, this study aimed to identify potential neurobiological mechanisms underlying these deficits and provide a scientific foundation for the development of targeted interventions aimed at improving social cognitive functioning in individuals with schizophrenia.

## 2 Materials and methods

### 2.1 Participants

The participants were recruited from Beijing Huilongguan Hospital between October 2020 and October 2024. Written informed consent was obtained from each participant prior to study participation. The study adhered to the principles of the Declaration of Helsinki and received approval from the ethics and institutional review boards of Beijing Huilongguan Hospital.

For the schizophrenia (SCZ) group, the inclusion criteria were as follows: (1) a diagnosis of schizophrenia according to the DSM-V criteria; (2) age between 18 and 60 years with at least 6 years of education; (3) voluntary participation by both the patient and their family members, with signed consent; and (4) a stable condition with the ability to communicate effectively. The exclusion criteria included: (1) intellectual disability or organic brain disorders; (2) severe withdrawal or impulsive behavior; (3) severe depression, anxiety, or substance abuse; and (4) serious physical illnesses or drug side effects impairing communication.

The healthy control (HC) group met the following criteria: (1) age between 18 and 60 years; (2) education level of junior high school or greater; (3) fluency in Mandarin; (4) clear speech without articulation disorders; (5) no family history of mental illness; (6) normal mental health status with no signs of anxiety or depression (confirmed through psychiatric interviews); and (7) normal scores on the Self-Rating Anxiety Scale and Self-Rating Depression Scale (<30 points).

### 2.2 Neuropsychological and psychopathological assessment

The evaluation of scales was conducted by a team of trained attending psychiatrists using standardized examination methods with high interrater reliability (intraclass correlation coefficient >0.8).

#### 2.2.1 Clinical symptom and social function assessment

The Temporal Experience of Pleasure Scale (TEPS) (14), Personal and Social Performance Scale (PSP) (15), Positive and Negative Symptom Scale (PANSS) (16), and Brief Negative Symptom Scale (BNSS) (17) were used to evaluate psychotic symptoms in the SCZ group. The PANSS is a standardized clinical interview comprising three subscales: positive symptoms (seven items), negative symptoms (seven items), and general psychopathology (16 items), totaling 30 items.

#### 2.2.2 Cognitive function assessment

The Chinese version of the MCCB (18), originally developed by the National Institute of Mental Health under the Measurement and Treatment Research to Improve Cognition in Schizophrenia (MATRICS) initiative, was used to assess cognitive function. The MCCB evaluates seven cognitive domains: speed of processing, working memory, verbal learning and memory, visual learning and memory, reasoning and problem-solving, attention/vigilance, and social cognition. It comprises 10 subtests.

#### 2.2.3 Facial emotion recognition

Facial emotion recognition in patients with schizophrenia is commonly assessed using standardized facial emotion images. This study utilized 42 facial emotion images representing seven emotions: anger, calmness, disgust, fear, happiness, sadness, and surprise, with six images per emotion. These static black-and-white photographs included an equal number of male and female faces. During the testing process, the participants completed a simulated test on a computer to ensure they understood the experimental task and procedure. In the formal test, facial images were presented randomly, and the participants were required to judge the emotion category and intensity of each image. Emotion intensity was rated using a bar scale ranging from 1 to 100.

#### 2.2.4 Voice emotion recognition

Voice emotion recognition research typically employs standardized evaluation paradigms. This study utilized 42 standardized voice emotion samples covering seven emotions: anger, calmness, disgust, fear, sadness, sarcasm, and surprise. Each emotion was represented by six voice segments—three from male and three from female speakers—at both high- and low-intensity levels. To assess participants’ engagement and attentiveness, a repeated voice segment was included as a quality control measure. During testing, participants sat in a quiet room wearing headphones and first completed a simulated test on a computer to familiarize themselves with the experimental task. In the formal test, the voice segments were presented randomly, and the participants were required to identify both the emotion category and intensity. Emotion intensity was rated on a 100-point scale. This method effectively evaluates voice emotion recognition deficits in individuals with schizophrenia, particularly differences in performance across emotional dimensions.

### 2.3 Statistical analysis

A diverse array of standard statistical methods was employed to analyze data and derive conclusions, including descriptive statistics and inferential statistics. Moreover, to compare features between the SCZ and HC groups, the Wilcoxon rank-sum test was employed to evaluate median differences, and the Benjamini–Hochberg procedure was used to adjust p-values, thereby controlling for false positives arising from multiple comparisons. The receiver operating characteristic (ROC) curves were used to define the sensitivity of clinical diagnostic tools. Area under the curve (AUC) analysis (DeLong variance estimation method for calculating statistics) were conducted. Principal component analysis (PCA) was conducted on the feature matrix, with the first two principal components utilized to generate a PCA plot, visually distinguishing the distribution of cases and controls in the feature space. Feature importance was determined based on the absolute value of each feature’s coefficient in the first principal component and subsequently ranked to identify the most influential features for disease diagnosis (19). Pairwise Pearson correlation coefficients were calculated, and a heatmap was generated to visually represent the strength of linear relationships between features. Network analysis was performed, with features represented as nodes and correlations as edges (20). The node color intensity reflected feature importance, while the edge width corresponded to the strength of the Pearson correlation, with only correlations >0.5 retained, to intuitively display the complex associations among features and their overall link to SCZ.

## 3 Results

### 3.1 Demographics, clinical characteristics, and cognitive function of the cohort

Exactly 106 patients with schizophrenia and 118 healthy controls were enrolled. We observed no significant difference in the basic demographic data between the patient and control groups. Regarding the severity of schizophrenia, the mean total PANSS score was 60.11 ± 20.57, BNSS score was 14.48 ± 7.34, and PSP score was 70.12 ± 13.48. The average course of the disease was 11.27 ± 8.87 years. The total scores of the TEPS and MCCB assessment of the patients were significantly lower than those of the healthy controls (p<0.01). Further details are provided in **Table 1**.

**Table 1:** General demographic data of the patients with schizophrenia and healthy controls.

Clinical informations		SCZ (n=106)	HCs (n=118)	p-value
Sex (male/female)		52/53	60/58	0.58
Age (years)		34.85 ± 9.96	32.60 ± 9.84	0.51
Years of education (years)		13.11 ± 3.08	12.73 ± 2.82	0.48
TEPS total score		77.83 ± 12.47	62.69 ± 10.16	<0.001
MCCB total score		39.30 ± 8.31	54.94 ± 7.17	<0.001
PANSS	Positive symptom score	13.14 ± 6.527	–	–
	Negative symptom score	14.93 ± 5.97	–	–
	General psychopathology score	30.12 ± 10.11	–	–
	PANSS total score	60.11 ± 20.57	–	–
BNSS	Anhedonia score	3.18 ± 3.17	–	–
	Distress score	1.15 ± 1.31	–	–
	Asociality score	2.81 ± 2.39	–	–
	Avolition score	2.50 ± 2.41	–	–
	Blunted affect score	3.36 ± 3.52	–	–
	Alogia score	2.06 ± 1.99		
	BNSS total score	14.48 ± 7.34	–	–
PSP	PSP total score	70.12 ± 13.48	–	–
Drug dosage	Chlorpromazine equivalents (mg)	590.14 ± 318.10	–	–
Disease course	Disease course (years)	11.27 ± 8.87	–	–

SCZ, schizophrenia group; HCs, healthy controls; TEPS, Temporal Experience of Pleasure Scale; MCCB, Measurement and Treatment Research to Improve Cognition in Schizophrenia (MATRICS) Consensus Cognitive Battery; PANSS, Positive and Negative Symptom Scale; BNSS, Brief Negative Symptom Scale; PSP, Personal and Social Performance Scale; chlorpromazine equivalents convert the drugs currently used by patients into chlorpromazine equivalents according to the drug dosage conversion standard. The highest equivalent of chlorpromazine in the SCZ group was 2400 mg/day, and the lowest equivalent was 75 mg/day.

### 3.2 Vocal and facial emotion recognition and intensity

The emotion recognition abilities between the SCZ and HC groups were compared. The results revealed significant differences in both facial and vocal emotion recognition. For facial emotion recognition, the SCZ group exhibited lower scores across various emotions, including anger, calmness, disgust, fear, happiness, sadness, and surprise. Specifically, the SCZ group had a mean facial score of 23.55 ± 7.10, compared to 31.86 ± 5.16 in the control group (p<0.001). In terms of vocal emotion recognition, the SCZ group also showed lower scores for emotions such as anger, calmness, disgust, fear, sadness, and surprise. The mean vocal score for the SCZ group was 18.64 ± 9.48, significantly lower than the control group’s score of 29.42 ± 5.01 (p<0.001). Notably, the SCZ group had higher facial intensity for happiness and surprise; however, their ability to recognize these emotions accurately was still impaired. These findings suggest that patients with schizophrenia have substantial difficulties in recognizing emotions from both facial expressions and vocal cues. Further details are provided in **Table 2**.

**Table 2:** Comparison of the emotion recognition and intensity between the SCZ and HC groups.

Emotions	Subjects	SCZ	HCs	p-value (FDR)
Anger	Facial Intensity	43.7 ± 15.29	41.92 ± 17.32	0.41
	Facial Score	3.15 ± 1.55	3.3 ± 1.38	0.36
	Vocal Intensity	53.53 ± 17.66	70.24 ± 15.17	<0.001
	Vocal Score	3.55 ± 1.69	4.84 ± 1.36	<0.001
Calmness	Facial Intensity	50.79 ± 16.13	50.33 ± 18.09	0.94
	Facial Score	4.36 ± 2.07	5.64 ± 0.75	<0.001
	Vocal Intensity	48.31 ± 15.75	49.17 ± 17.35	0.95
	Vocal Score	4.02 ± 1.89	5.39 ± 0.92	<0.001
Disgust	Facial Intensity	45.41 ± 16.38	45.92 ± 18.8	0.73
	Facial Score	2.77 ± 1.45	3.7 ± 1.55	<0.001
	Vocal Intensity	46.87 ± 15.23	49.37 ± 16.9	0.23
	Vocal Score	2.32 ± 1.23	3.69 ± 1.39	<0.001
Fear	Facial Intensity	53.33 ± 18.16	57.81 ± 17.95	0.034
	Facial Score	2.51 ± 1.46	4 ± 1.68	<0.001
	Vocal Intensity	46.18 ± 15.41	57.33 ± 16.83	<0.001
	Vocal Score	2.72 ± 1.7	4.52 ± 1.49	<0.001
Happiness	Facial Intensity	43.8 ± 16.97	33.48 ± 16.71	<0.001
	Facial Score	3.43 ± 1.66	4.99 ± 1.29	<0.001
Sadness	Facial Intensity	53.23 ± 18.52	57.27 ± 16.56	0.059
	Facial Score	4.38 ± 1.44	5.78 ± 0.57	<0.001
	Vocal Intensity	46.54 ± 15.02	53 ± 17.77	0.0036
	Vocal Score	3.14 ± 1.79	4.37 ± 1.46	<0.001
Satire	Vocal Intensity	47.31 ± 15.45	46.15 ± 17.22	0.56
	Vocal Satire Score	2.54 ± 1.45	3.45 ± 1.38	<0.001
Surprise	Facial Intensity	43.79 ± 17.06	36.72 ± 15.78	0.0013
	Facial Score	3.37 ± 1.76	4.52 ± 1.33	<0.001
	Vocal Intensity	47.7 ± 13.99	53.5 ± 19.65	0.019
	Vocal Score	3.35 ± 1.81	4.39 ± 1.31	<0.001
Total	Facial Intensity	47.64 ± 13.84	46.38 ± 13.66	0.72
	Facial Score	23.55 ± 7.1	31.86 ± 5.16	<0.001
	Vocal Intensity	55.79 ± 15.55	55.7 ± 13.29	0.82
	Vocal Score	18.64 ± 9.48	29.42 ± 5.01	<0.001

SCZ, schizophrenia group; HCs, healthy controls; FDR, false discovery rate.

In addition, we did not identify any correlation between sex, age, clinical course of disease, and drug equivalent with facial and vocal emotion recognition and intensity discrimination. Correlation analysis with PANSS and BNSS showed no significance (p>0.05).

### 3.3 Network analysis

**Figure 1:**
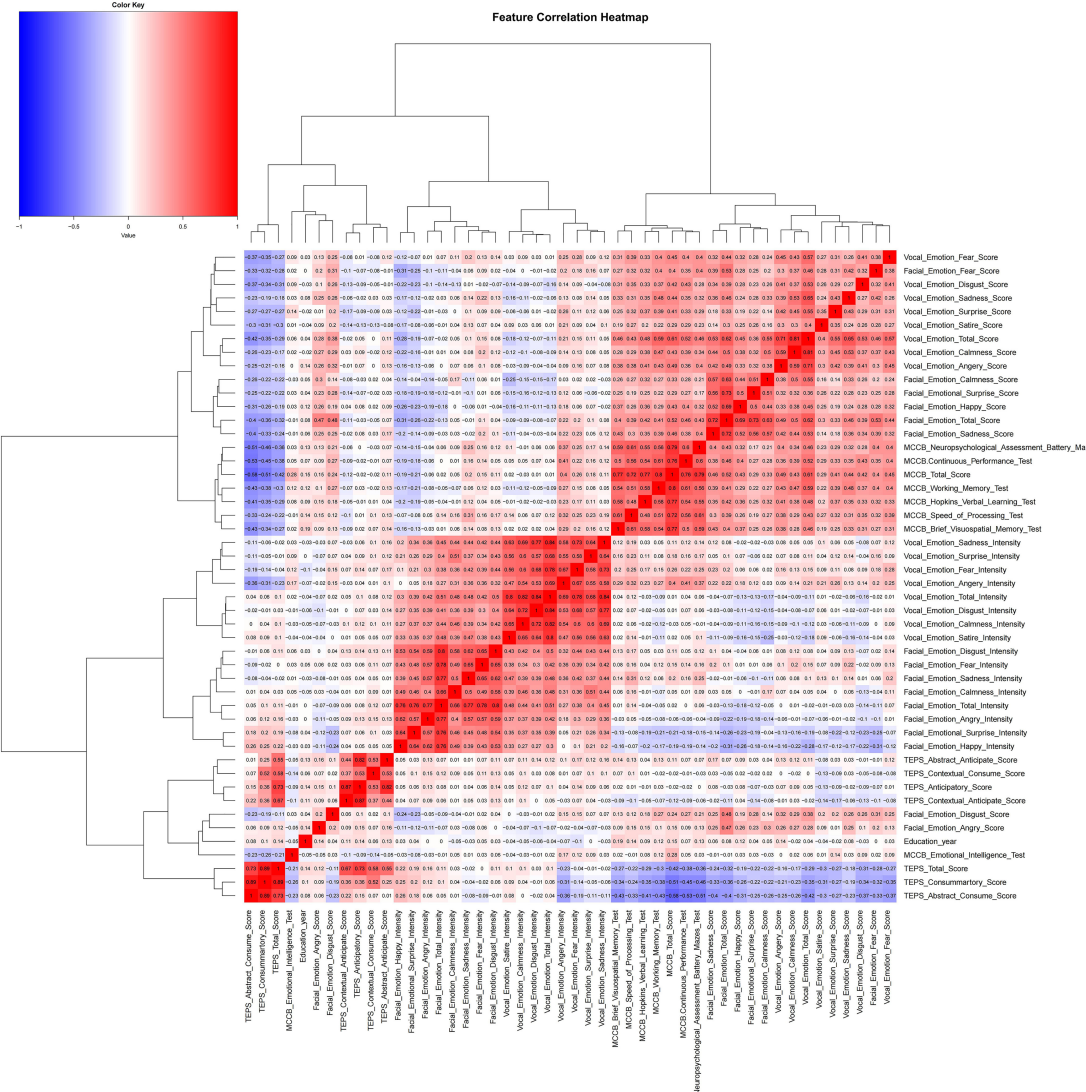
Feature correlation analysis of emotion recognition, cognitive function, and anticipatory pleasure measures. This heatmap displays the correlation matrix among various psychological, cognitive, and emotional features. The color intensity indicates the strength and direction of the correlation, with darker red representing stronger positive relationships and darker blue representing stronger negative relationships. TEPS, Temporal Experience of Pleasure Scale; MCCB, MATRICS Consensus Cognitive Battery.

Using a correlation coefficient graph (**Figure 1**), we assessed the pattern of recognition and intensity across the cohorts. The total score for emotional intensity and recognition was significantly correlated with specific emotional classifications (including anger, calmness, disgust, fear, sadness, satire, and surprise; Pearson’s correlation coefficient, r>0.9; p<0.001). Significant clusters were visualized in correlation plots for emotion recognition and intensity (**Figure 1**).

**Figure 2:**
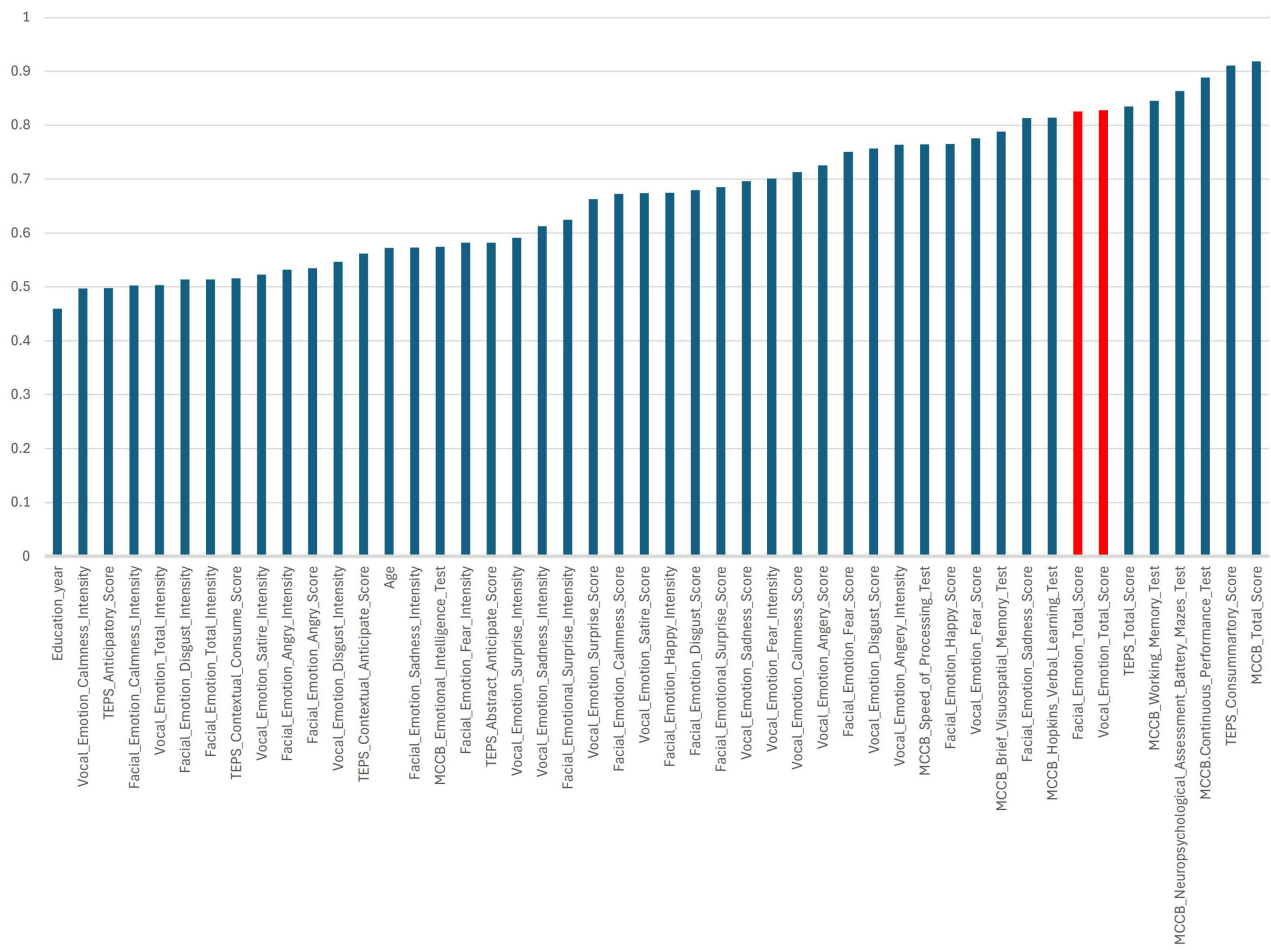
Feature network in schizophrenia with cognitive tests, emotion recognition, and pleasure-related domains. The figure shows a network visualization of feature relationships in patients with schizophrenia using correlation analysis and illustrates the interconnections among cognitive performance, anticipatory and consummatory pleasure, facial and vocal emotion recognition, and their relative importance in the analysis. Strong clustering among the MCCB cognitive tests suggests that the cognitive domains are highly interdependent. Subscales of the different cognitive and emotion recognition tests formed a distinct group, reflecting the strong interplay in the same category. Emotion recognition and intensity showed interrelations but also distinct pathways, indicating different neural processing mechanisms. Feature Importance (Node Size): Larger nodes indicate features with higher importance in the Principal Component Analysis (PCA) model. Correlation Strength (Edge Thickness): Thicker edges signify stronger relationships between variables, with correlation coefficients ranging from 0.6 to 0.8. Cluster Formation: Groups of interconnected nodes suggest shared variance among the features, revealing underlying cognitive-emotional mechanisms in patients with schizophrenia.TEPS, Temporal Experience of Pleasure Scale; MCCB, MATRICS Consensus Cognitive Battery.

The network analysis (**Figure 2**) highlighted significant interconnections among cognitive and affective features in patients with schizophrenia. Regarding the core cognitive functions, multiple tests from the MCCB, such as the Hopkins Verbal Learning Test, Neuropsychological Assessment Battery Mazes Test, and Brief Visuospatial Memory Test, exhibited strong correlations (r>0.9), underscoring their central role in assessing cognitive impairment in patients with schizophrenia. Importantly, the MCCB score was robustly associated with vocal and facial recognition domains (r>0.9), suggesting its key role in emotion recognition. In addition, we found that the voice and facial emotion intensity of the patients were independent of other clinical features, and the correlation with diseases was weakened (feature importance was approximately 0.05–0.10).

Regarding affective processing, the facial and vocal emotion recognition scores exhibited substantial intercorrelations (all r>0.6), indicating that deficits in different emotional dimensions, such as anger, fear, sadness, and surprise, were interconnected in the patients with schizophrenia. Furthermore, the intensity and recognition scores for both facial and vocal emotions were strongly correlated, implying a link between subjective emotional perception and objective performance.

In the context of anhedonia, scores from the TEPS revealed a close relationship with MCCB, reflecting the correlation between cognition and anhedonia in schizophrenia. Additionally, the TEPS scores were not significantly associated with emotion recognition or intensity, suggesting an isolated neural mechanism underlying reward processing and social-affective cognition in schizophrenia.

### 3.4 Emotion recognition-assisted clinical discrimination results

To enhance the assessment of schizophrenia diagnostic characteristics, we employed both traditional receiver operating characteristic analysis and PCA to quantify the importance of different clinical indicators in distinguishing patients from healthy individuals.

### 3.5 Diagnostic performance of the clinical indicators

**Figure 3:**
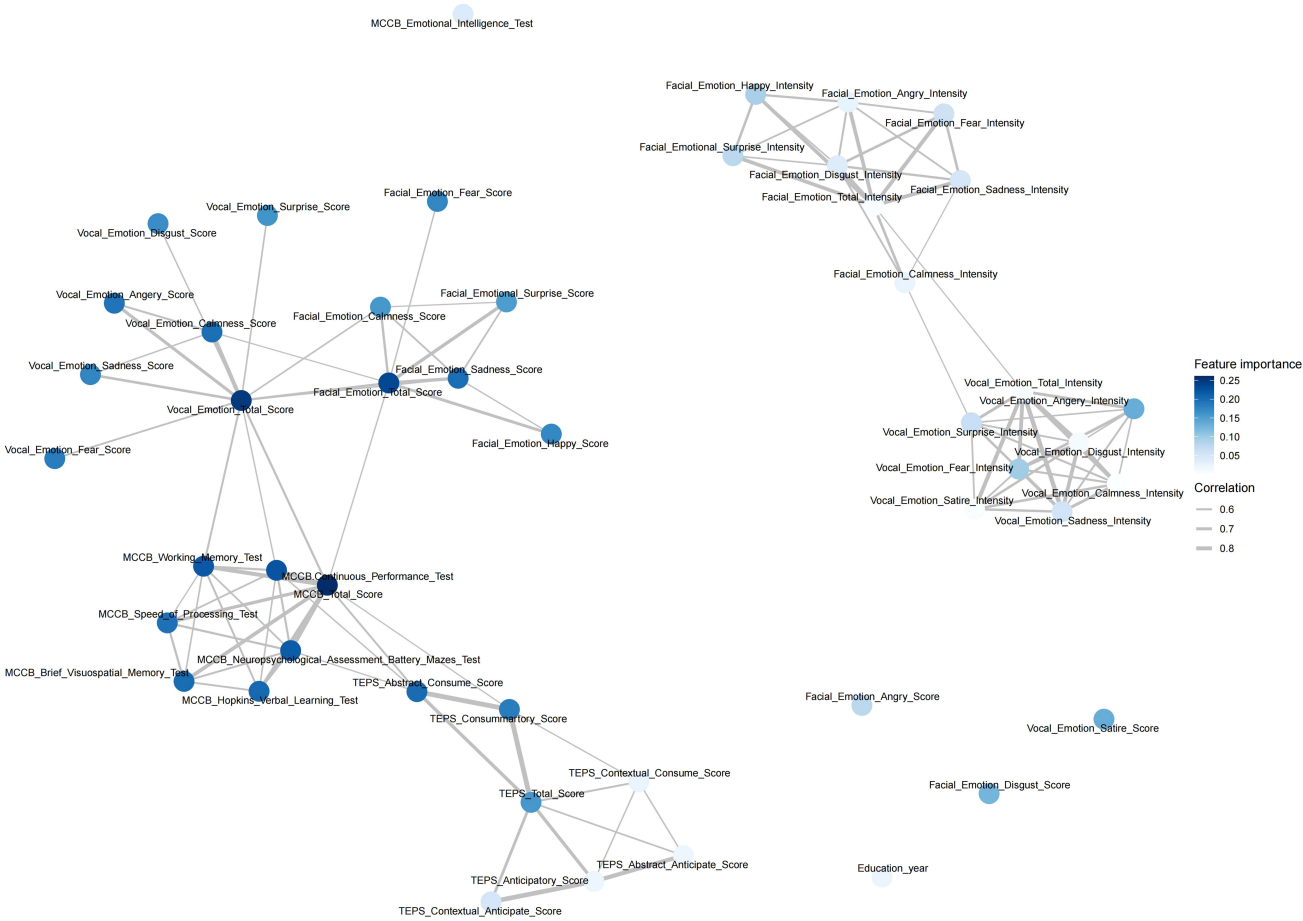
Clinical receiver operating characteristic curve (ROC) analysis. This analysis compared the diagnostic performance of cognitive function tools (MCCB tools), clinical emotion recognition tools (TEPS), and vocal emotion recognition tools in identifying schizophrenia. The results showed that emotion recognition performed well, with areas under the curve (AUCs) of 82.56% for vocal emotion recognition (p<0.01) and 82.82% for facial emotion recognition. In contrast, emotion intensity recognition had a relatively poor performance with an AUC of <70%, except for anger.

Based on patient and control phenotypes, we conducted diagnostic tests to evaluate the sensitivity and specificity of cognitive and emotional processing measures. The MCCB total score exhibited the highest diagnostic accuracy (area under the curve, AUC = 91.90%, p<0.01), followed by the TEPS scores (AUC = 91.13%, p<0.01). Emotion recognition abilities also contributed to the differentiation of patients with schizophrenia, with facial and vocal emotion recognition total scores demonstrating good predictive values (AUC_facial emotion recognition_ = 82.56%, AUC_vocal emotion recognition_ = 82.82%, p<0.01). However, vocal emotion intensity-based recognition was weak in distinguishing those with schizophrenia from healthy controls (AUC<60%), indicating its limited diagnostic utility (**Figure 3**).

### 3.6 PCA and group differentiation

**Figure 4:**
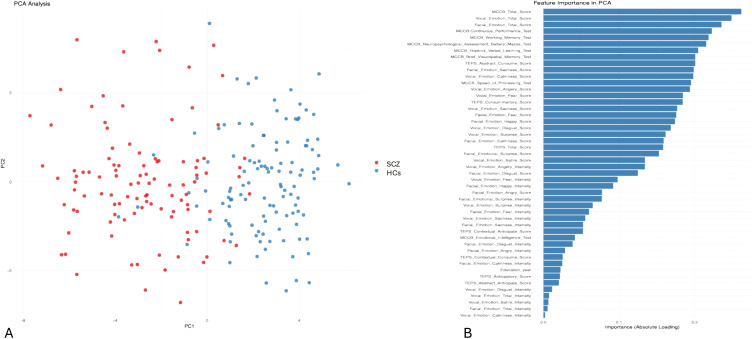
Principal component analysis (PCA) of cognitive and emotional processing in schizophrenia. **(A)** PCA plot illustrating the distribution of patients with schizophrenia (SCZ) and healthy controls (HCs) based on cognitive and emotional processing variables. The x-axis (PC1) represents the first principal component, capturing 23% of the variance among the analyzed features. The y-axis (PC2) represents the second principal component, capturing 18% of the variance. The HCs (blue) are more clustered, indicating greater homogeneity in cognitive and emotional processing. The patients (SCZ, red) display greater dispersion along PC1 and PC2, indicating higher interindividual variability. **(B)** This presents the importance of clinical and neuropsychological features in the PCA model used to differentiate patients with schizophrenia from HCs. The importance of each feature is indicated by its absolute loading value, reflecting its contribution to the principal components.

PCA revealed distinct clustering patterns between the SCZ and HC groups, highlighting key differences in cognitive function, anhedonia, and emotion recognition. The SCZ group exhibited greater dispersion along both the first (PC1, 23%) and second (PC2, 18%) principal components, reflecting high interindividual variability in these domains (**Figure 4**). In contrast, the HC group displayed a more compact and homogeneous distribution, suggesting more stable cognitive and emotional processing profiles. The PCA plot showed a clear separation between the SCZ and HC groups along PC1, indicating that the analyzed variables strongly contributed to diagnostic classification. PC1 accounted for a substantial portion of the total variance, with PC2 offering secondary but meaningful separation.

Overall, these findings underscore the diagnostic value of integrating cognitive function (MCCB), emotion recognition abilities, and TEPS for distinguishing patients with schizophrenia from healthy individuals. The significant variability within the SCZ group further emphasizes the heterogeneity of cognitive and emotional impairments in patients with schizophrenia.

## 4 Discussion

This study employed a comprehensive set of assessment tools, including facial and vocal emotion recognition tasks, cognitive function tests, and the TEPS, to systematically evaluate the performance of individuals with schizophrenia in emotion recognition, cognitive function, and hedonic experience. Additionally, network analysis and PCA were conducted to explore the relationships among various variables and identify potential clustering patterns. The integration of these methodologies enabled a multidimensional analysis of the interplay between emotional and cognitive impairments in schizophrenia. Furthermore, this study highlights that multimodal and multimethod evaluations may enhance the differentiation of symptoms and the characterization of schizophrenia-related features.

Cultural and ethnic factors may also influence cognition (21). Variations in emotional expression and perception across cultures may lead to differences in emotion intensity assessment and recognition in patients with schizophrenia (22). To date, systematic and integrated studies on emotion recognition in schizophrenia within Eastern cultural contexts remain scarce (3). This study provides a novel paradigm for investigating emotion recognition deficits based on a typical Eastern cultural framework, thereby filling an existing research gap.

Compared to the HC group, the SCZ group exhibited significant impairments in both vocal and facial emotion recognition. Additionally, their overall emotion discrimination ability was compromised, affecting not only the recognition of negative emotions (eg, anger, sadness, and fear) but also nonnegative emotions (eg, surprise, calmness, and happiness). Except for anger, the patients demonstrated a consistent pattern across vocal and facial emotion perception tasks. These findings are largely consistent with those of previous studies. Emerging evidence suggests these deficits may stem from impaired prefrontal-limbic circuit integration and dysregulated glutamatergic-GABAergic neurotransmission within sensory cortices (2, 3).

Importantly, this study supports the hypothesis that patients with schizophrenia exhibit a dissociation between explicit and implicit emotion processing at the behavioral level, as demonstrated through multimodal clinical testing (23). Explicit and implicit emotion recognition are fundamental concepts in understanding human emotional processing (24, 25). Explicit recognition refers to the conscious perception and expression of emotions, typically identified through distinct cues such as facial expressions, vocal intonations, and linguistic content. In contrast, implicit recognition involves unconscious or automatic emotional perception, reflected in a patient’s ability to assess emotional intensity (26). The patients in this study exhibited pronounced deficits in explicit vocal and facial emotion processing (ie, recognizing emotional content), whereas their implicit processing, particularly in assessing emotional intensity, was relatively preserved. Findings from the network analysis further reinforce this dissociative pattern, as different clusters of impairments in emotion recognition and intensity emerged in patients with schizophrenia.

This study also reveals differential impairments in the neural networks underlying explicit and implicit emotion processing in patients with schizophrenia. Explicit recognition typically engages higher-order cognitive functions, primarily involving the prefrontal and parietal cortices, which aligns with the observed strong correlations between emotion recognition performance and MCCB scores. Conversely, implicit recognition predominantly relies on limbic system structures, such as the amygdala and hippocampus, which play crucial roles in emotion processing (27). Notably, impairments in vocal emotion recognition were more pronounced than those in facial emotion recognition, both in diagnostic efficacy and in their association with cognitive deficits in schizophrenia. This suggests that functional abnormalities in auditory processing regions may be more prominent or develop earlier than structural and/or functional disruptions in core visual processing areas, such as the fusiform face area, occipitotemporal regions, and superior temporal sulcus (28).

In terms of clinical factors, this study did not find any significant correlations between emotion recognition deficits and demographic or clinical characteristics, including sex, age, disease duration, medication dosage, or even anhedonia severity. This finding aligns with that of some previous studies (29–31) and suggests that emotion cognition deficits remain relatively stable across different illness stages, regardless of the severity of acute symptoms. These deficits are unlikely to improve with the resolution of positive symptoms, suggesting that they are closely linked to the neurochemical and pathological mechanisms of schizophrenia itself (32), particularly specific neural circuits and information-processing pathways. Furthermore, although certain studies (33, 34) suggest that impairments in emotion recognition are closely linked to negative symptoms, including anhedonia, the experimental conditions and task formats vary across investigations. Some studies employ both motionless and animated visual stimuli, while others require patients to explicitly determine the emotions conveyed by the presented cues. These methodological discrepancies may contribute to the inconsistent findings regarding a patient’s ability to discern positive and negative emotions. The complexity of emotion perception in patients with schizophrenia suggests that multiple interacting factors may contribute to observed impairments, including variations in assessment methodologies.

### 4.1 Limitations

This study had several limitations. First, the relatively small sample size may affect the generalizability of the findings. Second, the assessment tools used primarily relied on subjective self-report measures. Future research should incorporate objective physiological indicators, such as neuroimaging techniques, to achieve a more comprehensive understanding of the mechanisms underlying emotion recognition deficits in schizophrenia. Moreover, future studies should increase the sample size, include patients at different disease stages, and employ longitudinal study designs to investigate how emotion recognition and cognitive impairments evolve over the course of schizophrenia.

## 5 Conclusion

Notably, emotion recognition deficits constitute a critical aspect of social cognitive impairment in schizophrenia, serving as a key determinant of functional outcomes and a symptom domain independent of positive and negative symptoms. This study systematically elucidates the interconnections among emotion recognition deficits, cognitive impairment, and anhedonia in schizophrenia, providing strong evidence for the interplay between social cognitive deficits and broader cognitive dysfunction. Additionally, this study establishes diagnostic characteristics and structured assessment tools, laying the foundation for precision interventions and personalized treatment strategies. The findings offer new directions for improving the social functioning of individuals with schizophrenia.

## Data Availability

The datasets presented in this study can be found in online repositories. The names of the repository/repositories and accession number(s) can be found below: https://www.bhlgh.com/.
